# Defined astrocytic expression of human amyloid precursor protein in Tg2576 mouse brain

**DOI:** 10.1002/glia.23550

**Published:** 2018-11-28

**Authors:** Tina Heiland, Ulrike Zeitschel, Maja A. Puchades, Peer‐Hendrik Kuhn, Stefan F. Lichtenthaler, Jan G. Bjaalie, Maike Hartlage‐Rübsamen, Steffen Roßner, Corinna Höfling

**Affiliations:** ^1^ Paul Flechsig Institute for Brain Research University of Leipzig Leipzig Germany; ^2^ Neural Systems Laboratory Institute of Basic Medical Sciences, University of Oslo Oslo Norway; ^3^ Institute of Pathology, Technical University of Munich Munich Germany; ^4^ Deutsches Zentrum für Neurodegenerative Erkrankungen (DZNE) Munich Germany; ^5^ Munich Cluster of Systems Neurology (SyNergy) Munich Germany; ^6^ Neuroproteomics, School of Medicine Klinikum rechts der Isar, Technical University of Munich Munich Germany; ^7^ Institute for Advanced Study Technical University of Munich Garching Germany

**Keywords:** Alzheimer's disease, astrocytes, human APP Swedish, microglia, oligodendrocytes, primary neuronal and glial cultures, Tg2576 mice

## Abstract

Transgenic Tg2576 mice expressing human amyloid precursor protein (hAPP) with the Swedish mutation are among the most frequently used animal models to study the amyloid pathology related to Alzheimer's disease (AD). The transgene expression in this model is considered to be neuron‐specific. Using a novel hAPP‐specific antibody in combination with cell type‐specific markers for double immunofluorescent labelings and laser scanning microscopy, we here report that—in addition to neurons throughout the brain—astrocytes in the corpus callosum and to a lesser extent in neocortex express hAPP. This astrocytic hAPP expression is already detectable in young Tg2576 mice before the onset of amyloid pathology and still present in aged Tg2576 mice with robust amyloid pathology in neocortex, hippocampus, and corpus callosum. Surprisingly, hAPP immunoreactivity in cortex is restricted to resting astrocytes distant from amyloid plaques but absent from reactive astrocytes in close proximity to amyloid plaques. In contrast, neither microglial cells nor oligodendrocytes of young or aged Tg2576 mice display hAPP labeling. The astrocytic expression of hAPP is substantiated by the analyses of hAPP mRNA and protein expression in primary cultures derived from Tg2576 offspring. We conclude that astrocytes, in particular in corpus callosum, may contribute to amyloid pathology in Tg2576 mice and thus mimic this aspect of AD pathology.

**Abbreviations**
AD: Alzheimer's disease
APP: amyloid precursor protein
DAB: 3,3′‐diaminobenzidine
GFAP: glial fibrillary acidic protein
GSTπ: glutathione‐S‐transferase π
hAPP: human APP
Iba1: ionized calcium binding adapter molecule 1
NeuN: neuronal nuclei antigen
TGFβ: transforming growth factor β

## 1 INTRODUCTION

Transgenic animal models are essential experimental tools to mimic aspects of Alzheimer's disease (AD) in order to study pathogenic mechanisms and to test therapeutic strategies. Typically, the transgenes are overexpressed and encode mutant forms of disease‐related human tau and amyloid precursor (APP) proteins or their processing enzymes (Bodendorf et al., 2002; Hsiao et al., 1996; Oakley et al., 2006; Oddo et al., 2003; Sturchler‐Pierrat et al., 1997). With regard to the amyloid pathology, a large magnitude of human APP (hAPP) constructs with mutations within the Abeta sequence or close to the beta cleavage site of the APP was used to generate transgenic mice that develop AD‐typical histopathology. These constructs differ with regard to the hAPP isoform used (APP695, APP751, APP770), the mutations introduced (Swedish, London, Dutch, Florida, Iberian, Arctic, Indiana, Iowa) and the promoter‐driving transgene expression (PDGF‐β, Thy1, CMV/β‐actin, CaMKII‐α, prion protein, neuron‐specific enolase) (Höfling et al., 2016). As a result, the onset and degree of amyloid pathology, neuronal and synaptic loss as well as changes in long‐term potentiation, neuronal network activity, and the degree of cognitive impairment differ substantially between the animal models established. Thus, it is indispensable to thoroughly characterize each model with regard to cell type and brain region‐specific transgene expression patterns.

The Tg2576 mouse model was established two decades ago (Hsiao et al., 1996) and had been made broadly available to the scientific community. These mice overexpress hAPP695 carrying the Swedish double mutation KM670/671NL under control of the hamster prion protein promoter and develop amyloid plaques at 11–13 months of age predominantly in neocortex and hippocampus (Hartlage‐Rübsamen et al., 2011; Hsiao et al., 1996; Kawarabayashi et al., 2001). Interestingly, synaptic spine loss in the hippocampal CA1 region (Lanz, Carter, & Merchant, 2003), changes in hippocampal long‐term potentiation (Jacobsen et al., 2006), hypersynchrony of neuronal network activity (Shah et al., 2016), and cognitive impairment (King & Arendash, 2002) were reported at 4–6 months of age, indicating the importance of soluble neurotoxic Abeta assemblies or of intracellular APP C‐terminal fragments (Xu, Fitzgerald, Nixon, Levy, & Wilson, 2015) present before amyloid plaque pathology.

Another important aspect to explain variability in neuropathology and behavioral disturbances among APP‐transgenic mouse lines is the cell type and neuron type‐specific expression of hAPP. Both, the hamster prion protein promoter‐driving hAPP expression in FVB/N and Tg2576 mice (Hsiao et al., 1995; Hsiao et al., 1996) and transgenic hAPP expression itself were reported to be neuron‐specific (Irizarry, Locascio, & Hyman, 2001; Irizarry, McNamara, Fedorchak, Hsiao, & Hyman, 1997). More specifically in brain, the endogenous hamster prion protein was found to be expressed by neurons of the hippocampus, septum, caudate putamen, thalamic nuclei, and dorsal root ganglia cells (Bendheim et al., 1992). In general, similar patterns of hAPP transgene expression and amyloid pathology are observed in Tg2576 mice (Hartlage‐Rübsamen et al., 2018; Hsiao et al., 1996; Irizarry et al., 2001; Kawarabayashi et al., 2001). So far, to the best of our knowledge, no glial expression of hAPP in Tg2576 brain was demonstrated. However, using a novel antibody that differentiates between endogenous mouse and hAPP (Höfling et al., 2016), we here report the presence of nonneuronal cells immunoreactive for hAPP in a brain region‐specific manner. To identify the glial cell types expressing the transgene, double immunofluorescent labelings of hAPP with cell type‐specific marker proteins were performed in brains of 3‐ and 18‐month‐old Tg2576 mice and analyzed by confocal laser scanning microscopy. In addition, primary neurons and glial cells derived from Tg2576 mice were analyzed for hAPP mRNA and protein expression by RT‐qPCR and immunocytochemistry, respectively.

Our data indicate that not only neurons, but also astrocytes may contribute to amyloid pathology in the Tg2576 mouse model of AD.

## 2 MATERIALS AND METHODS

### 2.1 Experimental animals

Breeding pairs of the hAPP‐transgenic mouse line Tg2576 were kindly provided by Dr. Karen Hsiao (University of Minnesota). Tg2576 mice were maintained on C57BL/6xSJL background in which transgene expression is driven by the hamster prion protein promoter (Hsiao et al., 1996). For the characterization of hAPP expression, transgenic Tg2576 mice and their wild type littermates were examined at the postnatal age of 3 and 18 months. Animals were housed in groups of 3–5 animals per cage and separated by sex with food and water ad libitum at 23 °C for 12 hr day/12 hr night cycles in cages that contained red plastic houses (Tecniplast, Hohenpeißenberg, Germany) and shredded paper flakes to allow nest building. At the age of 6 weeks, the transgenicity of the animals was tested by polymerase chain reaction of tail DNA, as described elsewhere (Hsiao et al., 1996). All experimental protocols were approved by Landesdirektion Sachsen, license T28/16 and all methods were carried out in accordance with the relevant guidelines and regulations.

### 2.2 Tissue preparation

For immunohistochemistry, adult mice (3 and 18 months of age) were sacrificed by CO_2_ inhalation and transcardially perfused with 50 ml of 0.9% saline followed by perfusion with 50 ml of 4% paraformaldehyde in PB (0.1 M; pH 7.4). The brains were removed from the skull and postfixed by immersion in the same fixative overnight at 4 °C. After cryoprotection in 30% sucrose in 0.1 M PB for 3 days, coronal sections (30 μm) were cut at the level of basal forebrain (Bregma 1.10 mm) and hippocampus (−1.80 mm) on a sliding microtome and collected in 0.1 M PB containing 0.025% sodium azide. The level of the basal forebrain was selected because of the prominent existence of nonneuronal cells, for example, oligodendrocytes and astrocytes in the corpus callosum white matter. The hippocampal coronal cutting level was chosen according to previously described findings of senile Abeta plaques in the hippocampus and neocortex of Tg2576 mice.

### 2.3 Immunohistochemistry

#### 2.3.1 Single labeling hAPP immunohistochemistry

All immunohistochemical procedures were performed on free‐floating brain sections. Sections were pre‐treated with 1% H_2_O_2_ in 60% methanol for 1 hr to abolish endogenous peroxidase activity. Unspecific staining was blocked in TBS containing 5% normal donkey serum and 0.3% Triton‐X100 before incubating the brain sections with the primary antibodies against hAPP (rat anti‐hAPP, clone 1D1, 1:4) at 4 °C overnight. The following day, sections were subsequently incubated with secondary, biotinylated donkey antibodies directed against rat IgG (Dianova; 1:1,000) for 60 min at room temperature, followed by the ABC method, which composed incubation with complexed streptavidin—biotinylated horseradish peroxidase. Incubations were separated by washing steps (3 × 5 min in TBS). Binding of peroxidase was visualized by incubation with 4 mg 3,3′‐diaminobenzidine (DAB) and 2.5 μl H_2_O_2_ per 5 ml Tris buffer (0.05 M; pH 7.6) for 3–5 min, resulting in brown labeling.

The specificity and applicability of the 1D1 antibody has been extensively characterized recently (Höfling et al., 2016). It allows differentiation between endogenous mouse and transgenic human APP and binds an extracellular N‐terminal hAPP epitope between amino acids 40 and 64. Thus, it does not detect Abeta peptides, but rather the hAPP and can be applied for immunocytochemistry, immunohistochemistry, immunoprecipitation and Western blot as well as FACS analyses (Höfling et al., 2016). Importantly, using Western blot analyses, robust hAPP bands at approximately 100 kDa were present in brain tissue homogenates of APP‐transgenic Tg2576, 3xTg and I5 mice, whereas bands were neither detected at molecular weights of Abeta peptides or C‐terminal APP fragments nor in wild type mouse brain tissue (Supporting Information Figure S1).

#### 2.3.2 Double immunofluorescence histochemistry

To reveal the cell type‐specific staining generated by the rat monoclonal anti‐hAPP antibody 1D1 in Tg2576 mouse brain, double immunofluorescent labelings in combination with antibodies directed against specific markers of neurons (mouse anti‐NeuN; Chemicon; 1:1,000), astrocytes (rabbit anti‐GFAP; Dako; 1:500), microglia (rabbit anti‐Iba1; Wako; 1:500), and oligodendrocytes (rabbit anti‐GSTπ; Merck; 1:500) were performed. Brain sections were incubated with cocktails of primary antibodies at 4 °C overnight. On the next day, sections were washed three times with TBS and were then incubated with cocktails of Cy3‐conjugated donkey anti‐rat (1:200; Dianova) and Cy2‐conjugated donkey anti‐rabbit or donkey anti‐mouse (1:200; Dianova) for 60 min at room temperature. This procedure resulted in red fluorescent labeling of hAPP‐positive cells and in green fluorescent labeling of the respective neuronal or glial cell types. After washing, sections were mounted onto glass slides and coverslipped.

### 2.4 Microscopy

#### 2.4.1 Light microscopy

Tissue sections of 3‐month‐old and of 18‐month‐old wild type and transgenic Tg2576 mice with single labeling by DAB for hAPP expression were examined with an Axio‐Scan.Z1 microscope connected with a Colibri.7 light source and an Axiocam 506 (Carl Zeiss, Göttingen, Germany). Images were digitized by means of ZEN 2.3 software and exported with the NetScope program (Net‐Base Software GmbH, Freiburg, Germany).

#### 2.4.2 Confocal laser scanning microscopy

Confocal laser scanning microscopy (LSM 510, Zeiss, Oberkochen, Germany) was performed to allow allocation of hAPP to neurons, astrocytes, microglia, and oligodendrocytes, respectively in Tg2576 mouse brain. For Cy2‐labeled cell type marker proteins (green fluorescence), an argon laser with 488 nm excitation was used and emission from Cy2 was recorded at 510 nm applying a low‐range band pass (505–550 nm). For Cy3‐labeled hAPP (red fluorescence), a helium‐neon‐laser with 543 nm excitation was applied and emission from Cy3 at 570 nm was detected applying high‐range band pass (560–615 nm). Photoshop CS2 (Adobe Systems, Mountain View, CA) was used to process the images obtained by light and confocal laser scanning microscopy with minimal alterations to brightness, sharpness, color saturation, and contrast.

### 2.5 Cultivation of Tg2576 primary neuronal and glial cells

#### 2.5.1 Primary neurons

The preparation and cultivation of neural primary cells was conducted according to a modified method from Löffner, Lohmann, Walckhoff, Walter, and Hamprecht (1986) as described in Hartlage‐Rübsamen et al. (2015). Briefly, fetuses of Tg2576 mice rom gestation day 16 of Tg2576 mice were prepared, individually genotyped and cultured. Typically, 50% of the offspring was hAPP‐transgenic and 50% wild type. Neurons were dissociated into single cells by triturating the brains by means of a pipette and passing the cell suspensions through sterile nylon meshes (20 μm). Suspensions were then grown in seeding medium (DMEM/Ham's F‐12 supplemented with 5% fetal horse serum (FHS) and 1% penicillin–streptomycin–neomycin (PSN) antibiotic mixture) in T25 cell culture flasks (for mRNA analyses) and on poly‐l‐lysine‐coated glass coverslips in 24‐well culture plates (for immunocytochemistry), respectively. The cells were cultured at 37 °C in a humidified atmosphere containing 5% CO_2_. On the following day, the seeding medium was exchanged by neuronal medium (DMEM/Ham's F‐12 supplemented with 5% FHS, 1% PSN, 1% N‐2 supplement, and 30% astrocyte conditioned medium). On day 3, cells in the T25 cell culture flasks were rinsed with ice‐cold, sterile PBS, covered with 2.5 ml TRIzol reagent (Invitrogen, Carlsbad, CA) and stored at −20 °C for cell lysis and later RNA extraction. The cells on coverslips in the 24‐well plates were fixed with 4% PFA for 10 min and stored in TBS (0.1 M, pH 7.4) at 4 °C for immunofluorescent labeling.

#### 2.5.2 Primary glial cells

For the cultivation of glia‐rich primary cell cultures, newborn Tg2576 mice were sacrificed by decapitation and brains as well as tail tips for genotyping were collected. The brains were dissociated into single cells by triturating through pipettes of decreasing width and passed through sterile nylon meshes (150 μm). After resuspending the cells with 5 ml astrocyte medium (DMEM supplemented with 10% fetal bovine serum [FBS], and 1% PSN), 5 ml of each cell suspension were transferred into a T25 cell culture flask and 4 × 500 μl into a 24‐well plate containing sterile glass coverslips, respectively. The cells were cultured at 37 °C in a humidified atmosphere of 5% CO_2_. The medium was renewed on the third day after seeding and henceforth, once a week.

Between Culture Days 12 and 20, primary *microglial cells* were separated from the primary astrocytes by subjecting the suspensions to vibrations in a shaking incubator (SI500, Stuart) at 260 rpm and 37 °C for 30 min. The cell suspensions were then transferred to new T25 cell culture flasks (5 ml) and 24‐well plates (4 × 500 μl) containing sterile glass coverslips, respectively, and were cultured for 3 days under the conditions mentioned above.

For the separation of primary *oligodendrocytes*, the glia‐rich primary cultures were incubated in 5 ml oligodendroglia medium (DMEM supplemented with 1% PSN and 1% N‐2 supplement) in a shaking incubator at 220 rpm and 37 °C for 18 hr. Afterward, the cell suspensions were split and filled in precoated T25 cell culture flasks (5 ml) and 24‐well cell culture plates (4 × 500 μl) containing precoated, sterile glass coverslips, respectively. Primary oligodendrocytes were cultured for 3 days under the conditions mentioned above.

Subsequently, all primary glial cell cultures in the T25 cell culture flasks were rinsed with ice‐cold, sterile PBS, covered with 2.5 ml TRIzol reagent (Invitrogen) and stored at −20 °C for cell lysis and RNA extraction at a later time point. The primary glia cultures in the 24‐well cell culture plates were fixed with 4% PFA for 10 min and stored in TBS (0.1 M, pH 7.4) at 4 °C awaiting immunofluorescent labeling.

### 2.6 Immunocytochemistry

To determine the cell type‐specific expression of transgenic hAPP in primary neuronal and glial cells using the fixed primary cells grown on glass coverslips, double immunofluorescent labelings with 1D1 and cell type‐specific antibodies were performed as described for immunohistochemistry on brain sections. Finally, the coverslips were air‐dried, embedded in entellan/toluene on microscopic slides and stored at 4 °C in the dark.

In addition, double labeling with all cell type‐specific antibodies was performed in each subculture of neuronal and glial primary cells, to examine the identity and purity of the respective primary cultures (see Supporting Information Figure S2).

### 2.7 RNA isolation and RT‐qPCR

To analyze the respective mRNA expression of transgenic hAPP in neuronal and glial primary cell cultures of transgenic Tg2576 mice, reverse transcription quantitative polymerase chain reaction (RT‐qPCR) was performed. RNA of cultivated wild type and Tg2576 neurons, astrocytes, microglia, and oligodendroglia was isolated using the TRIzol RNA isolation protocol (Chomczynski & Mackey, 1995). Quality and concentration of RNA was analyzed with the photometer NanoDrop 2000 at wavelengths 260 and 280 nm. The total RNA obtained from the isolation of primary neuronal and glial cell cultures of transgenic Tg2576 mice and their wild type littermates was reverse transcribed into complementary DNA (cDNA) and amplified in an one‐step reaction using the OneStep RT‐PCR kit (Qiagen, Hilden, Germany) and a Rotor‐Gene™ 6000 Real‐Time PCR System (Corbett Research, Sydney, Australia).

The following forward and reverse primers for hAPP and the housekeeping gene Cyclophilin A (CycA) were diluted in RNase free water resulting in a concentration of 10 μM: hAPP‐fw: GTGGCATTCTTTTGGGGCTG, hAPP‐rev: GAACCTGGTCGAGTGGTCAG (product length 108 bp), CycA‐fw: AAGACTGAATGGCTGGATGG, CycA‐rev: TTACAGGACATTGCGAGCAG (product length 237 bp). For the RT‐qPCR, a master mix containing RNase‐free water (2.5 μl), 5× OneStep RT‐PCR Buffer (2.0 μl), 5× Q‐solution (2.0 μl), dNTP mix (10 mM each; 0.4 μl), OneStep RT‐PCR Enzyme Mix (0.4 μl), forward and reverse primers (0.6 μl each) and SYBR Green (1:200) (1.0 μl) was prepared for each primer pair.

To generate a standard curve for each primer pair, a dilution series (1:5) was prepared yielding six different RNA concentrations of RNA isolated from the complete left brain hemisphere of a transgenic Tg2576 mouse, starting at 500 ng/μl. Furthermore, RNA isolated from the complete left brain hemisphere of a wild type C57/Bl6 mouse was diluted to obtain a concentration of 10 ng/μl. RNA samples isolated from primary cultures were diluted in preparation for five solutions of each genotype containing 10 ng/μl and denaturized in a thermal cycler at 70 °C for 5 min.

A volume of 9.5 μl of the master mix was mixed with 0.5 μl of the respective denaturized RNA. The RT‐qPCR was carried out in a Rotor‐Gene™ 6000 Real‐Time PCR System according to the following protocol: hold stage 1 (50 °C, 1,800 s, 95 °C, 900 s), cycling stage (denaturation 95 °C, 10 s, annealing 60 °C, 45 s, elongation 72 °C, 45 s, melting 84 °C, 10 s) and hold stage 2 (72 °C, 15 s). The cycling stage covered 30 cycles.

The specificity of the PCR products was evaluated by melting curves which were generated by a rise in temperature to 84 °C. Furthermore, the specificity was tested by separating all samples in agarose gel electrophoresis (2% agarose in TAE [1×] containing 0.01% GelRed) to control correct product length.

## 3 RESULTS

The inspection of Tg2576 mouse brain slices labeled with the antibody 1D1 revealed the expression of hAPP by numerous neurons as described previously (Höfling et al., 2016). To allow for a comprehensive evaluation of the hAPP immunoreactivity in Tg2576 mouse brain, we provide a link to access a serial hAPP staining, where every fourth coronal brain section was stained and scanned (http://cmbn‐navigator.uio.no/navigator/filmstrip_viewer.html?publicOnly=true&entityType=block&entityId=4247#). This link to the virtual microscopy viewer Navigator3 allows interactive zooming and panning of high‐resolution images. The series has been registered to the Allen brain mouse atlas available from: http://connectivity.brain-map.org/) using the QuickNII software tool (Puchades, Csucs, Ledergerber, Leergaard, & Bjaalie, 2017). This link shows the same sections with their custom atlas overlay, adjusted for angle deviations. (http://cmbn‐navigator.uio.no/navigator/filmstripzoom/filmstripzoom_lite.html?atlas=300000&series=4247&preview=ABAMousev2Preview.png).

**Figure 1:**
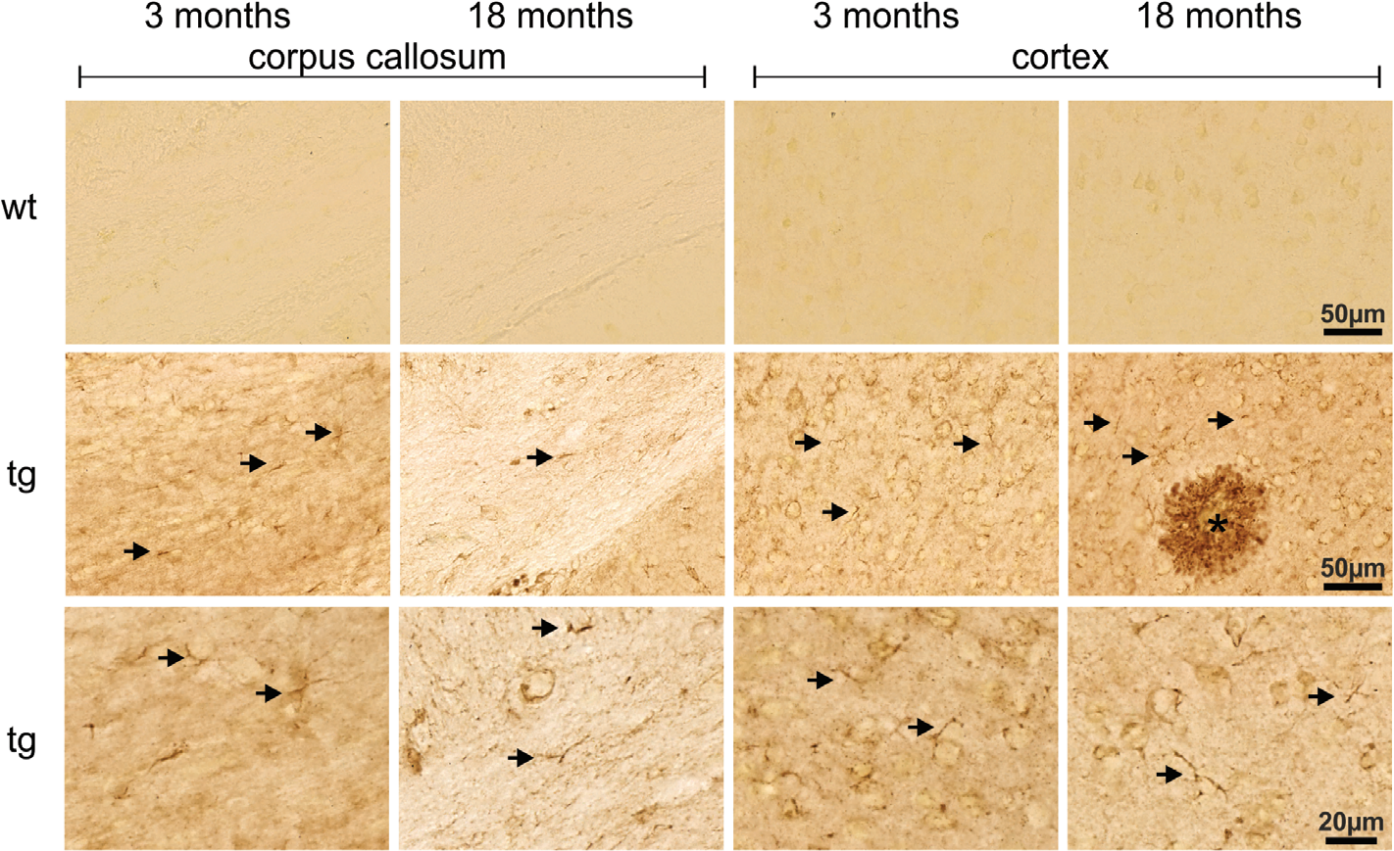
Immunohistochemical detection of hAPP in corpus callosum and cortex of young and aged Tg2576 mice (tg) and wild type littermates (wt) as indicated. Immunoreactivity for hAPP is absent in wild type brain sections demonstrating the specificity of the 1D1 antibody. Although the majority of the 1D1 labeling arises from neurons, glial structures (arrows) are also hAPP‐immunoreactive. This is displayed at higher magnification in the bottom images (arrows)

The typical appearance of hAPP immunoreactivity in parietal cortex and corpus callosum of 3‐ and 18‐month‐old Tg2576 mouse brain is shown in Figure 1. Note the membranous labeling of cells with neuronal size and shape in young and aged Tg2576 mouse brain in parietal cortex and corpus callosum and the complete absence of hAPP immunoreactivity in wild type mouse brain sections. In addition to the neuronal labeling, hAPP was detected in smaller, branched nonneuronal cells as shown at high‐magnification images in Figure 1 (arrows). This glial‐like hAPP immunoreactivity is more prominent in corpus callosum compared with parietal cortex, which might be due to the higher density of neuronal cell bodies in cortex. There are no obvious differences in neuronal and glial‐like hAPP immunoreactivity between 3‐ and 18‐month‐old Tg2576 mice. However, hAPP immunoreactivity was found to be also present in amyloid plaque‐associated dystrophic neurites of 18‐month‐old transgenic mice (Figure 1).

**Figure 2:**
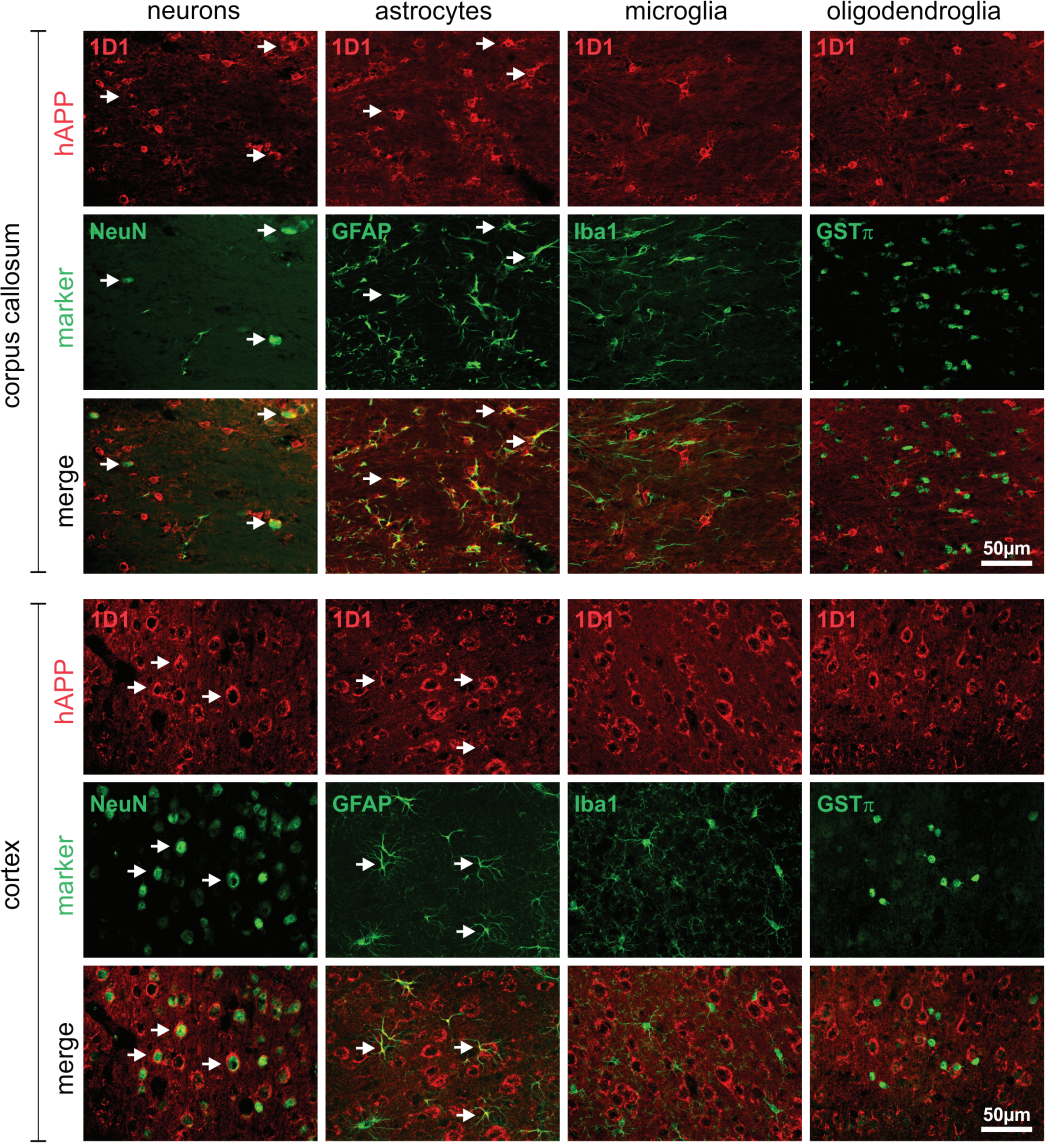
Cell type‐specific expression of hAPP in brains of 3‐month‐old Tg2576 mice. The hAPP in corpus callosum and cortex was visualized using the antibody 1D1 and detection with secondary Cy3‐conjugated antibodies (red fluorescence) in combination with marker proteins for neurons (NeuN), astrocytes (GFAP), microglia (Iba1), and oligodendrocytes (GSTπ) detected with Cy2‐conjugated secondary antibodies (green fluorescence). Note the frequent co‐localization of hAPP with neurons and astrocytes (arrows)

To identify the origin of the hAPP immunoreactive glial processes, double immunofluorescent labelings with cell type‐specific marker proteins were performed and evaluated by laser scanning microscopy. In corpus callosum and neocortex of 3‐month‐old Tg2576 mice without amyloid pathology, hAPP frequently co‐localized with the neuronal marker NeuN, but neither with the microglial marker Iba1 nor with the oligodendrocyte marker GSTπ (Figure 2). The neuronal expression of hAPP was validated by double labeling of hAPP with the neuronal marker MAP2 (Supporting Information Figure S3A). However, a substantial proportion of GFAP‐immunoreactive astrocytes in corpus callosum and some astrocytes in cortex were found to be hAPP‐immunoreactive (Figure 2).

**Figure 3:**
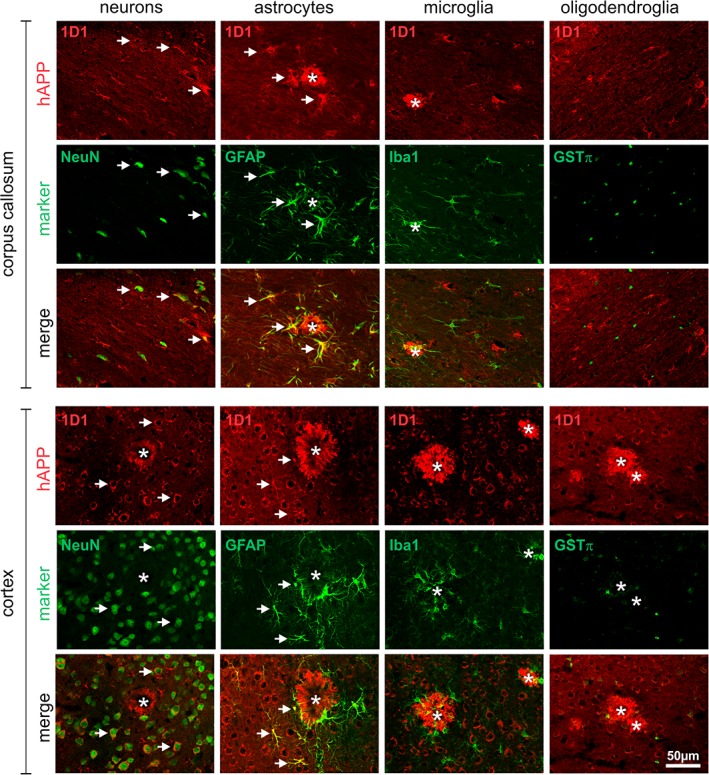
Cell type‐specific expression of hAPP in brains of 18‐month‐old Tg2576 mice. The hAPP in corpus callosum and cortex was visualized using the antibody 1D1 and detection with secondary Cy3‐conjugated antibodies (red fluorescence) in combination with marker proteins for neurons (NeuN), astrocytes (GFAP), microglia (Iba1), and oligodendrocytes (GSTπ) detected with Cy2‐conjugated secondary antibodies (green fluorescence). Note the frequent co‐localization of hAPP with neurons and astrocytes (arrows) and the absence of hAPP immunoreactivity in amyloid plaque‐associated reactive astrocytes in cortex

Also in 18 month‐old Tg2576 mouse brain, hAPP was localized to neurons and to astrocytes in corpus callosum and cortex, but not to microglia and oligodendroglia (Figure 3). Interestingly, in corpus callosum both resting astrocytes distant from amyloid plaques and reactive astrocytes in proximity to amyloid plaques displayed hAPP immunoreactivity, whereas in cortex only nonreactive astrocytes close to or distant from amyloid plaques expressed hAPP immunoreactivity (Figure 3). The APP expression by astrocytes was also confirmed by using two alternative antibodies (6E10 and 22C11) in combination with GFAP antibodies (Supporting Information Figure S3B). However, it should be noted that endogenous mouse APP might have contributed to these signals as 6E10 and 22C11 do not differentiate as well between mouse and transgenic hAPP as does the antibody 1D1.

**Figure 4:**
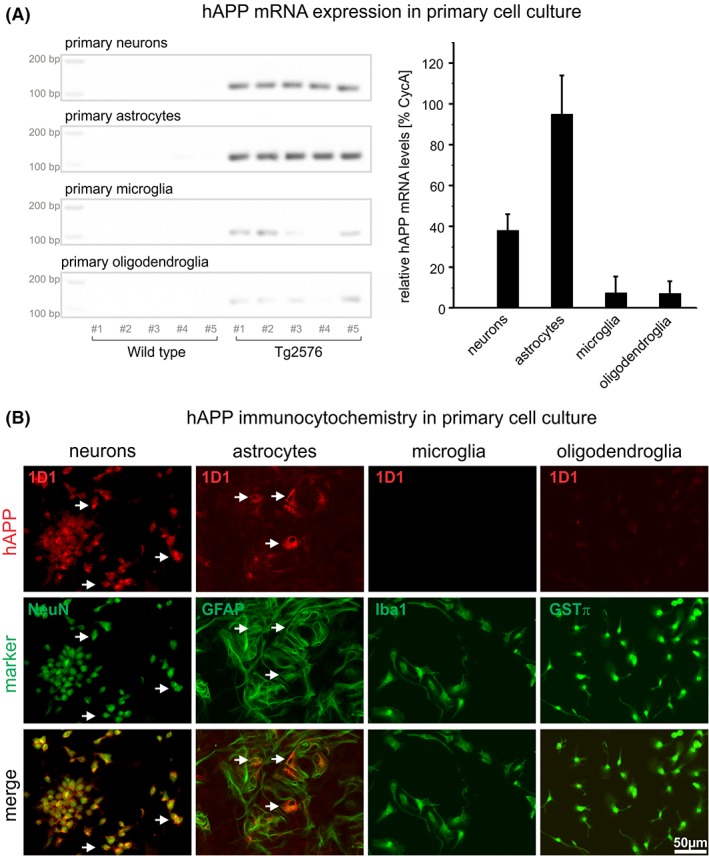
Specific detection of hAPP mRNA (A) and protein (B) in Tg2576 primary neurons and glial cells by RT‐qPCR and immunocytochemistry, respectively. (A) In neuronal and astrocytic and to a much lesser extent in microglial and oligodendroglial cultures of Tg2576 mice hAPP mRNA is detected, whereas in the corresponding cultures of wild type mice no hAPP mRNA was present. This is consistent with the specificity of primer pairs used for hAPP versus mouse APP. On the left hAPP PCR products separated on agarose gels from different cell types are shown. The diagram on the right shows the CycA‐normalized quantification of hAPP mRNA by RT‐qPCR in the respective cell types. (B) Primary neuronal and astrocytic cultures of Tg2576 mice display hAPP immunoreactivity (arrows), which is absent from microglial and oligodendroglial Tg2576 cultures

The robust astrocytic hAPP immunoreactivity raises the question whether hAPP produced by and released from neurons is taken up by astrocytes or whether it is expressed by these glial cells. To address this issue, primary neuronal and glial cell cultures from Tg2576 mice were established and analyzed for hAPP mRNA and protein expression. Brains of newborn mice were prepared, genotyped, and individually cultured. With regard to genomic DNA, approximately 50% of the offspring was hAPP transgenic and 50% wild type. Figure 4a shows representative examples of hAPP PCR products from five different neuronal, astrocytic, microglial, and oligodendroglial cultures of wild type and Tg2576 mice separated on agarose gels (left) and the CycA‐normalized quantification of hAPP PCR products in the respective cell types (right). In neurons and astrocytes derived from transgenic mice, significant hAPP mRNA expression was detected by RT‐qPCR. In contrast, in microglial and oligodendroglial cultures of hAPP transgenic mice, very low hAPP transcript levels were detected. In neuronal and glial cultures established from wild type mice, no hAPP mRNA was detected, demonstrating the specificity of the primers for hAPP (Figure 4a).

Immunocytochemical labeling of neuronal and glial cultures of wild type origin with the hAPP‐specific antibody did not result in any labeling (not shown). Neurons and astrocytes of transgenic mice were found to be immunoreactive for hAPP (Figure 4b). This is in agreement with immunohistochemical labelings of brain slices, and supportive of de novo synthesis of hAPP by astrocytes, rather than uptake of neuron‐derived hAPP. In microglial and oligodendroglial cultures, no hAPP immunoreactive cells were detected. The minor hAPP mRNA levels detected in these cultures may arise from the presence of some astrocytes (Supporting Information).

## 4 DISCUSSION

The Tg2576 mouse model developed by Hsiao et al. (1996) is one of the most frequently used animal models to investigate aspects of amyloid pathology, accompanying gliosis as well as functional and behavioral consequences of the formation of pathogenic Abeta assemblies. To completely understand pathogenic mechanisms in this mouse model, a thorough analysis of the brain region and cell type‐specific transgene expression is indispensable. A recently developed rat monoclonal antibody differentiating between mouse and human APP allowed for such an analysis and had already been used by us to demonstrate alterations in transgene expression patterns of different hAPP transgenic mouse lines and rats (Höfling et al., 2016). Subsequently, we were able to reveal a spatial correlation between transgene expression and the formation of amyloid plaques (Hartlage‐Rübsamen et al., 2018).

Here, in serial sections immunohistochemically labeled for hAPP, we observed stained cellular structures that did not display neuronal morphology. Glial hAPP expression was already mentioned for FVB/N mice expressing the same promoter or hAPP695s that were constructed, but the glial cell type and brain region‐specific occurrence were not reported (Hsiao et al., 1995). Therefore, we here analyzed the cell type‐specific hAPP expression in young and aged Tg2576 mouse brain. By immunohistochemical double labelings of hAPP and cell type‐specific marker proteins, we demonstrate the expression of hAPP by astrocytes in the corpus callosum and to a much lesser extent in neocortex of young Tg2576 mice without amyloid plaque pathology. In contrast, neither microglial cells nor oligodendrocytes were found to express hAPP.

To rule out that glial immunoreactivity arises from uptake of hAPP produced by neurons, primary glial cultures from Tg2576 offspring were analyzed for hAPP mRNA and protein expression. These experiments confirmed gene expression of hAPP in astrocytes but not in microglial cells and oligodendrocytes.

Our results are corroborated by another study which recently demonstrated that primary cultured astrocytes from Tg2576 mice express transgenic hAPP and secrete Abeta peptides into the culture medium decreasing the number of readily releasable synaptic vesicles and excitatory synaptic transmission in co‐cultured neurons (Katsurabayashi et al., 2016). Moreover, the expression of hAPP in astrocytic Tg2576 cultures and its processing into amyloidogenic fragments were found to be stimulated by addition of oligomeric and fibrillary Abeta preparations, most likely through the stimulation of astrocytic BACE1 expression (Zhao, O'Connor, & Vassar, 2011). However, in brain tissue of aged Tg2576 mice with robust amyloid pathology, we did not observe an induction of hAPP expression in reactive astrocytes in proximity to Abeta plaques. The reason for this discrepancy is not known but could be based on the application of defined Abeta preparations in cell culture experiments, whereas, in brain tissue, astrocytes are exposed to a broad spectrum of hAPP cleavage products and Abeta peptide variants.

Nevertheless, the stimulus‐dependent capacity of reactive astrocytes to express APP (Avila‐Muñoz & Arias, 2015; Siman, Card, Nelson, & Davis, 1989) as well as the APP processing enzyme BACE1 (Hartlage‐Rübsamen et al., 2003) and components of the γ‐secretase complex (Nadler et al., 2008) indicate a potential astrocytic contribution to amyloid pathology. Indeed, intracellular Abeta was detected in astrocytes of AD cortex (Akiyama et al., 1999).

As hAPP expression in Tg2576 mouse brain is driven by the hamster prion protein promoter, its expression pattern reflects to a large extent on this promoter activity and may differ drastically from that of the endogenous mouse APP promoter. Hamster prion protein mRNA and protein were reported to be predominantly expressed by neurons, but also by astrocytes, particularly in corpus callosum and optical nerve of hamster and rat (Moser, Colello, Pott, & Oesch, 1995) as well as in cultured mouse primary astrocytes (Lima et al., 2007). The translation of the endogenous prion protein mRNA in astrocytes was shown to be robust, but not upregulated during reactive astrogliosis in mouse brain (Jackson, Krost, Borkowski, & Kaczmarczyk, 2014), which might partly explain the lack of hAPP induction in reactive astrocytes in proximity to Abeta plaques of Tg2576 mice.

Also in human brain, nonneuronal expression of APP mRNA has been demonstrated (Golde, Estus, Usiak, Younkin, & Younkin, 1990) and multiple pro‐inflammatory cytokines have been shown to upregulate APP expression and Abeta secretion in human astrocyte cultures (Blasko et al., 2000; Brugg et al., 1995). Astrocytic cell lines and human astrocytes respond with increased APP expression when exposed to TGFβ (Amara, Junaid, Clough, & Liang, 1999; Burton, Liang, Dibrov, & Amara, 2002; Gray & Patel, 1993), a cytokine which is associated with AD pathogenesis (Luedecking, DeKosky, Mehdi, Ganguli, & Kamboh, 2000). This implies that TGFβ increases Abeta levels in the AD brain by inducing APP upregulation in astrocytes (Frost & Li, 2017). In the neuroinflammatory context of AD, reactive astrocytes express higher levels of APP than resting astrocytes and, therefore, could produce more Abeta and contribute to amyloid pathology to a greater extent. Thus, with regard to the induction of hAPP expression by reactive astrocytes, the Tg2576 mouse model clearly differs from the human condition in AD.

Another important aspect of astrocytic hAPP expression in Tg2576 brain is the more prominent hAPP immunoreactivity in callosal compared with neocortical astrocytes (see also filmstrip images at Section 3). It is well‐known that astrocytes compose the most abundant and diverse type of glial cells in the brain (Lundgaard, Osório, Kress, Sanggaard, & Nedergaard, 2014). During development astrocytes adapt during development to the needs of the surrounding tissue which could be a reason for the different density of astrocytes in different brain regions (Emsley & Macklis, 2006; Wang & Bordey, 2008). It is unknown which factors are decisive for the adaption of the specific morphology, but there are distinct morphological differences between astrocytes in grey and white matter which result in two prominent types: protoplasmic and fibrous astrocytes, respectively. White matter astrocytes have smaller cell bodies and their processes are aligned with myelinated fibers. This diversity of morphology is accompanied by different protein expression profiles. CD44, for example, is predominantly expressed by fibrous astrocytes in white matter (Kaaijk, Pals, Morsink, Bosch, & Troost, 1997). On the physiological level, corpus callosum astrocytic calcium waves were shown to propagate by a mechanism independent of gap junctional coupling (Schipke, Boucsein, Ohlemeyer, Kirchhoff, & Kettenmann, 2002), whereas in neocortical grey matter calcium wave propagation does depend on functional astrocytic gap junctions (Haas et al., 2006). Astrocytes also express glutamate transporters for clearing the extracellular space of this neurotransmitter. The synapse density‐normalized glutamate transporter activity is significantly higher in white matter than in grey matter (Hassel, Boldingh, Narvesen, Iversen, & Skrede, 2003). In addition, the capacity for glutamate metabolism to glutamine is higher in white matter than in grey matter astrocytes (Goursaud, Kozlova, Maloteaux, & Hermans, 2009) supporting the hypothesis that glutamate clearance might be more important in white matter to avoid excitotoxicity due to glutamate overload. In the Tg2576 mouse model, an age‐dependent elongation of magnetic resonance transverse relaxation time (T2) values in the corpus callosum but a significant T2 decrease in grey matter cortex and hippocampus was reported (Kara et al., 2015). This noninvasive measure is indicative of loss of corpus callosum integrity and was confirmed by histological analyses of demyelination, gliosis and amyloid‐plaque deposition in the corpus callosum (Kara et al., 2015).

In addition to neurons and glial cells, cultured vascular muscle cells from brains of Tg2576 mice were shown to produce hAPP and to deposit Abeta intracellularly, indicating that cerebrovascular amyloid in Tg2576 mice is at least partially of nonneuronal origin and that vascular smooth muscle cells are a source of these amyloid deposits (Frackowiak, Miller, Potempska, Sukontasup, & Mazur‐Kolecka, 2003). This is also consistent with the expression of endogenous prion protein by muscle cells (Bendheim et al., 1992).

Together, we here demonstrate that hAPP expression in brains of Tg2576 mice is not restricted to neurons but has a significant astrocytic component, particularly in corpus callosum white matter. These astrocytes may, therefore, contribute to amyloid pathology in Tg2576 mouse brain making them targets for experimental pharmacological intervention studies. Interestingly, astrocytes in the APP/PS1 AD mouse model were also shown to be implicated in disease mechanisms by contributing to neuronal‐glial network dysfunction, which can be ameliorated by P2Y1 receptor antagonists (Reichenbach et al., 2018). Our data underline the importance of investigating cell type‐specific transgene expression in hAPP transgenic mouse lines.

## Supporting information

Supplementary Information
