# Supplementary material for: Defined astrocytic expression of human amyloid precursor protein in Tg2576 mouse brain
Source: Glia. 2018 Nov 28;67(2):393–403. doi: 10.1002/glia.23550 (PMC6588085; doi:10.1002/glia.23550)
Supplement: Supplementary file 1 — Supplementary Information [file GLIA-67-393-s001.docx]

**Supplementary Information; Heiland et al.**

Specificity of the 1D1 antibody for hAPP

The specificity of the rat monoclonal 1D1 antibody to detect hAPP, but not mouse APP was demonstrated by Western blot analysis. Briefly, PBS soluble fractions were prepared from neocortex of 18-month-old mouse brain samples in PBS containing complete protease inhibitor cocktail (Roche) followed by a clarifying centrifugation step at 15,000 x g. The remaining pellet was solubilized in 1% Triton in STE buffer (150 mM NaCl, 50 mM Tris, 2 mM EDTA) followed by a clarifying spin at 15,000 x g. Samples were boiled for 5 minutes at 95°C in non-reducing Laemmli buffer and used for non-reducing SDS PAGE. 20 µg total protein per sample were loaded onto 10% polyacrylamide gels, separated by electrophoresis and transferred onto nitrocellulose membranes by overnight blotting. The membranes were incubated with 1D1 (3 µg/ml) antibodies for 2 hours at room temperature, followed by incubation with secondary, horseradish peroxidase-conjugated goat anti-rat antibody and chemiluminescent visualisation.

Compared to the APP-transgenic mouse lines 3xTg (Oddo et al., 2003) and I5 (Mucke et al., 2000), the strongest hAPP signal was detected in Tg2576 brain homogenate, whereas no signal was detected using wild type mouse brain homogenate (**Suppl. Fig. 1**). Overall, only bands at molecular weight above 100 kDa were detected, consistent with the binding of 1D1 to the full length APP molecule. No signals were detected at the molecular weight of Abeta peptides or C-terminal fragments A4-CT or P3-CT.


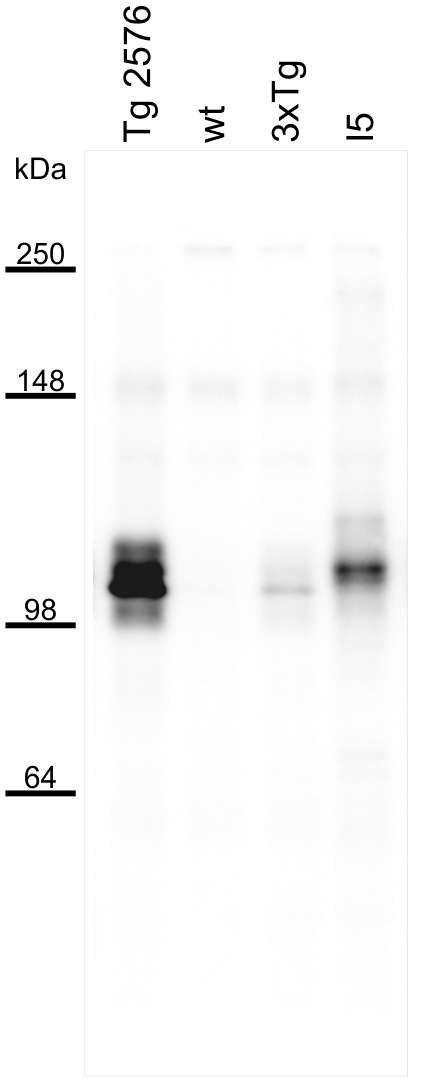


**Suppl. Fig. 1:** The 1D1 antibody was tested in brain homogenates of wild type mice (wt) and APP-transgenic mouse models Tg2576, 3xTg and I5 for its specificity towards hAPP by Western blot analysis. 1D1 detected bands at a molecular weight above 100 kDa only in brain homogenates of APP-transgenic but not in wt mice.

Purity of primary neuronal and glial cell cultures

In order to characterize the purity of the mouse primary neuronal and glial cultures used for the analyses of hAPP expression, double fluorescent immunocytochemistry was performed to detect cell type-specific markers in all cell types. For immunocytochemistry, primary antibodies from different species were used (**Suppl. Table 1**).

**Supplementary Table 1:** Primary antibodies used for immunocytochemical labellings.

| **antibody** | **source (clone)** | **concentration [µg/µL]** | **dilution** | **supplier** | **catalogue #** |
| --- | --- | --- | --- | --- | --- |
| anti-NeuN | mouse (A60) | 1.0 | 1:200 | Merck | MAB377 |
| anti-GFAP | mouse (G-A-5) | 1.0 | 1:2,000 | Sigma | G3893 |
| anti-GFAP | rabbit | 2.9 | 1:2,000 | Dako | Z0334 |
| anti-Iba1 | guinea pig | N/A | 1:1,000 | Synaptic Systems | 234004 |
| anti-Iba1 | rabbit | 0.5 | 1:500 | Wako | 019-19741 |
| anti-GSTπ | rabbit | N/A | 1:2,000 | Merck | AB8902 |

These antibodies were combined as cocktails for double labelling of neuronal and glial markers on primary cells grown on coverslips (**Suppl. Table 2**).

**Supplementary Table 2:** Combinations of primary antibodies used for immunocytochemical double labellings of primary neuronal and glial cell cultures.

| **Cell type analyzed** | **neuronal marker** | **astroglial marker** | **microglial marker** | **oligodendr. marker** |
| --- | --- | --- | --- | --- |
|  | mouse anti-NeuN | rabbit anti-GFAP |  |  |
| **Neurons** | mouse anti-NeuN |  | rabbit anti-Iba1 |  |
|  | mouse anti-NeuN |  |  | rabbit anti-GSTπ |
|  | mouse anti-NeuN | rabbit anti-GFAP |  |  |
| **Astrocytes** |  | mouse anti-GFAP |  | rabbit anti-GSTπ |
|  |  | rabbit anti-GFAP | guinea pig anti-Iba1 |  |
|  | mouse anti-NeuN |  | rabbit anti-Iba1 |  |
| **Microglia** |  | mouse anti-GFAP | guinea pig anti-Iba1 |  |
|  |  |  | guinea pig anti-Iba1 | rabbit anti-GSTπ |
|  | mouse anti-NeuN |  |  | rabbit anti-GSTπ |
| **Oligodendroglia** |  | mouse anti-GFAP |  | rabbit anti-GSTπ |
|  |  |  | guinea pig anti-Iba1 | rabbit anti-GSTπ |

Employing these immunocytochemical methods, the high purity of all primary cell cultures was demonstrated (**Suppl. Fig. 2**).


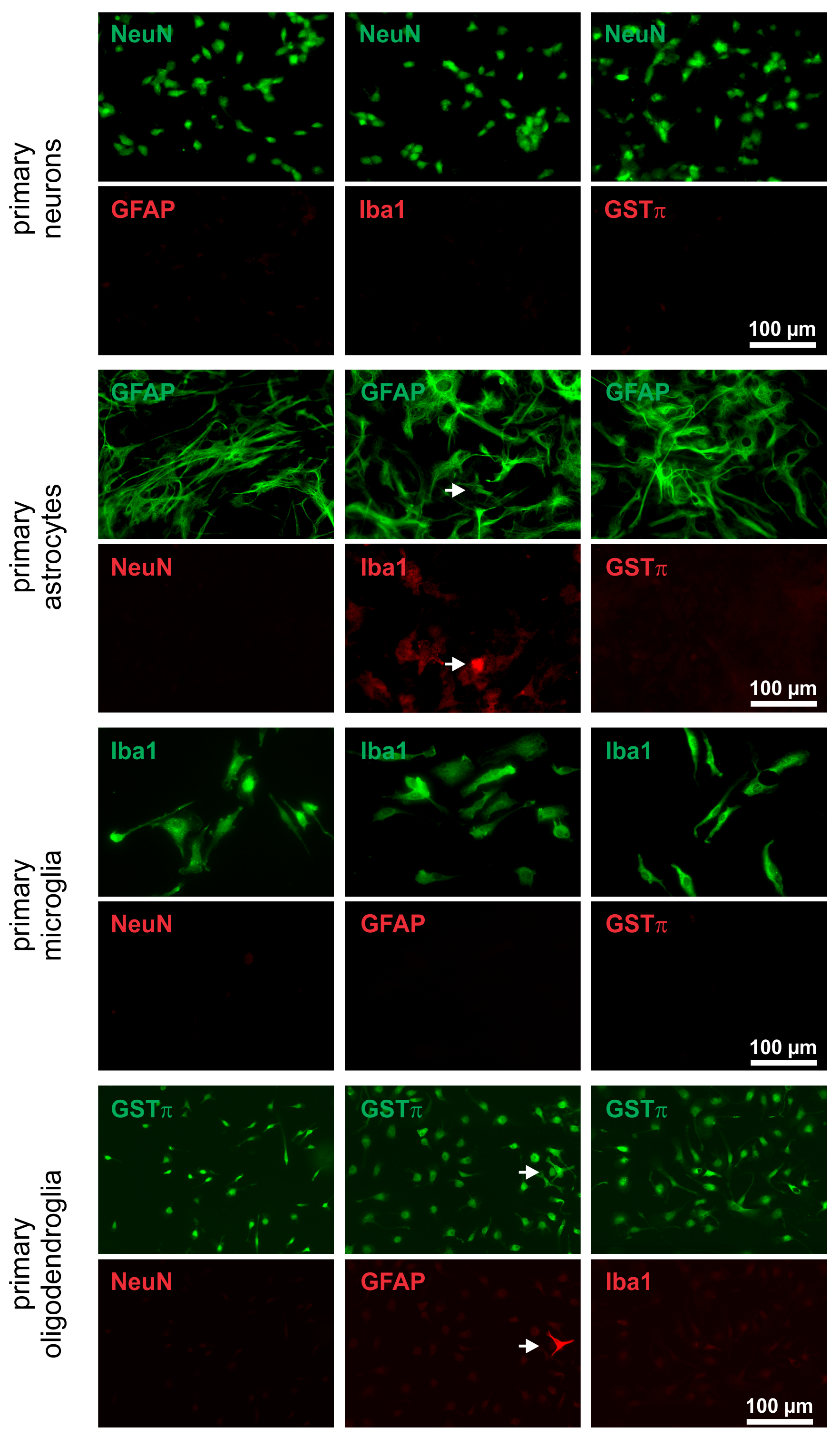


**Suppl. Fig. 2:** Purity of primary neuronal and glial cultures analyzed by immunocytochemistry using the neuronal marker NeuN, the astrocytic marker GFAP, the microglial marker Iba1 and the oligodendroglial marker GSTπ. In all cases, the respective cell types are highly enriched.

Validation of neuronal and astrocytic hAPP expression in Tg2576 mouse brain

In order to validate the predominant neuronal expression of hAPP shown in the main document by double labelling with NeuN, we here additionally show a double immunofluorescent labelling with the neuronal marker MAP2 (**Suppl. Fig. 3A**). As expected, there was a significant co-localization of hAPP and MAP2. In addition, in order to validate the astrocytic expression of APP in Tg2576 mouse brain, double immunofluorescent labellings of GFAP with the N-terminal APP antibody 22C11 and with the human-specific APP antibody 6E10 were performed. In both cases a subpopulation of astrocytes was identified as being hAPP-immunoreactive, confirming the data obtained with the 1D1 antibody (**Suppl. Fig. 3B**). It should be noted, however, that 22C11 also recognizes endogenous mouse APP and that 6E10 binds to the Abeta domain of APP. Therefore, in these combinations a contribution of mouse APP and human Abeta, respectively, to the immunohistochemical labelling cannot be ruled out.


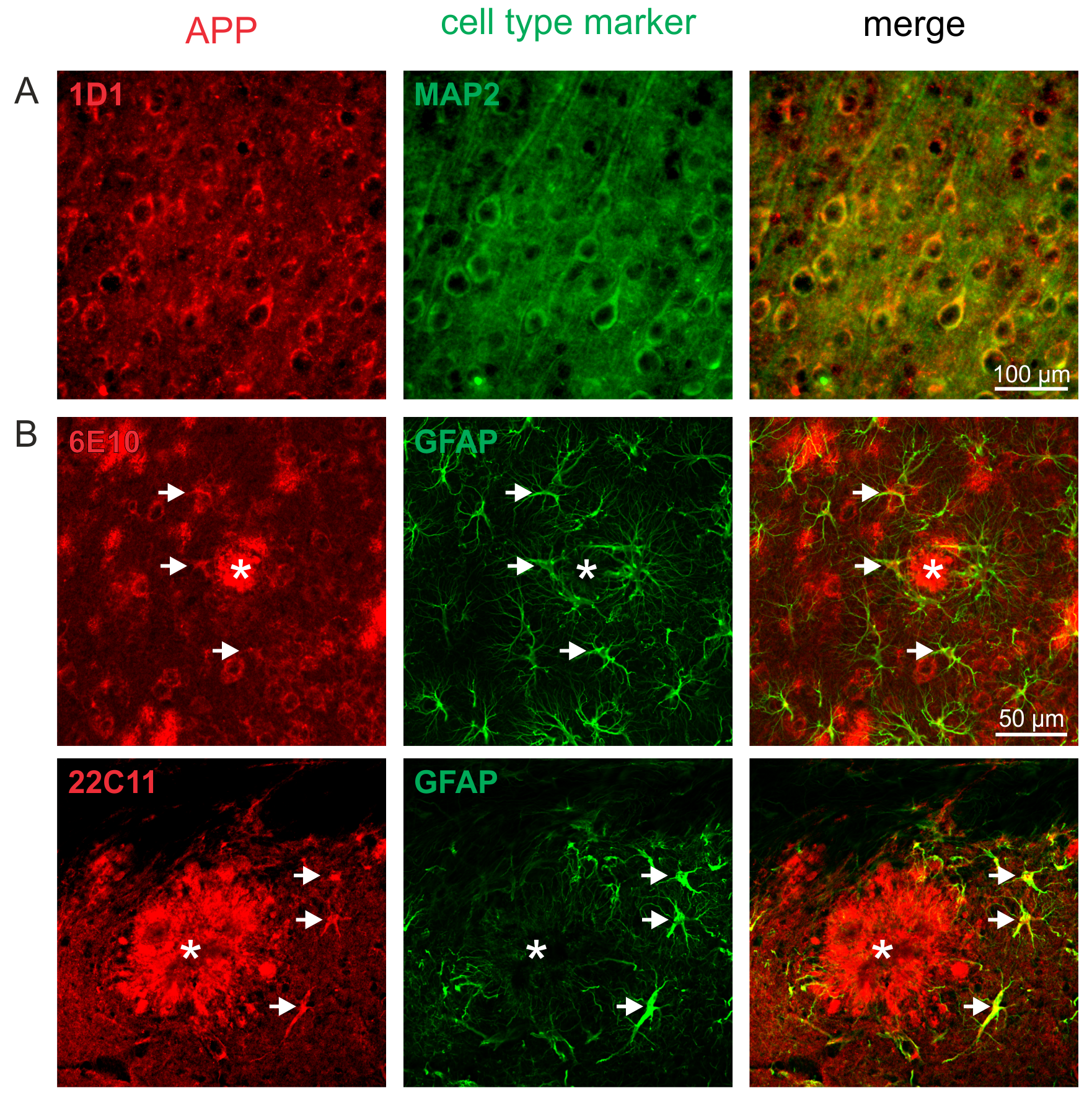


**Suppl. Fig. 3:** **(A)** Co-localization of hAPP detected with the antibody 1D1 (red fluorescence) with the neuronal marker MAP2 (green fluorescence). **(B)** Demonstration of astrocytic APP immunoreactivity (arrows) by using the mouse anti-APP antibodies 6E10 and 22C11 (red fluorescence) in combination with rabbit anti-GFAP antibodies (green fluorescence) in brain sections of 18-month-old Tg2576 mouse brain. The asterisks indicate the position of amyloid plaques.

**Suppl. References:**

Oddo, S., et al., 2003. Triple-transgenic model of Alzheimer's disease with plaques and tangles: intracellular Abeta and synaptic dysfunction. Neuron 39, 409-421.

Mucke, L., et al., 2000. High-level neuronal expression of abeta 1-42 in wild-type human amyloid protein precursor transgenic mice: synaptotoxicity without plaque formation. J. Neurosci. 20, 4050-4058.
